# Development of a list of competencies and entrustable professional activities for resident physicians during death pronouncement: a modified Delphi study

**DOI:** 10.1186/s12909-022-03149-5

**Published:** 2022-02-22

**Authors:** Takaomi Kessoku, Yu Uneno, Yuka Urushibara-Miyachi, Kiyofumi Oya, Akihiko Kusakabe, Atsushi Nakajima, Noritoshi Kobayashi, Yasushi Ichikawa, Mitsunori Miyashita, Manabu Muto, Masanori Mori, Tatsuya Morita

**Affiliations:** 1 Department of Palliative Medicine, Yokohama City University Hospital, 3-9 Fukuura, Kanazawa-ku, Yokohama, 236-0004 Japan; 2 Department of Gastroenterology and Hepatology, Yokohama City University Graduate School of Medicine, 3-9 Fukuura, Kanazawa-ku, Yokohama, 236-0004 Japan; 3 Department of Therapeutic Oncology, Graduate School of Medicine, Kyoto University, Kyoto, 606-8507 Japan; 4 Faculty of Medicine, Kyoto University, Yoshida konoe-cho, Sakyo-ku, Kyoto, 606-8501 Japan; 5 Department of Transitional and Palliative Care, Aso Iizuka Hospital, Fukuoka, 820-8505 Japan; 6 Department of General Medicine, Yokohama City University School of Medicine, Yokohama, 236-0004 Japan; 7 Department of Oncology, Yokohama City University Hospital, Yokohama, 236-0004 Japan; 8 Department of Palliative Nursing, Health Sciences, Tohoku University Graduate School of Medicine, Sendai, 980-8575 Japan; 9 Department of Palliative and Supportive Care, Seirei Mikatahara General Hospital, Hamamatsu, 433-8558 Japan

**Keywords:** Delphi, Competencies, Death pronouncement, Entrustable professional activities, Resident physicians

## Abstract

**Background:**

The appropriate delivery of death pronouncements potentially affects bereaved families’ wellbeing positively. Although younger physicians need to learn the competencies and entrustable professional activities (EPAs) to conduct death pronouncement independently, both of which have not been clarified. Therefore, this study aimed to develop a list of competencies and EPAs necessary for death pronouncement practice, which resident physicians need to acquire by the end of their residency training (postgraduate year 2).

**Methods:**

An anonymous modified Delphi study was conducted with a panel of 31 experts. The experts were invited online from general wards in hospitals with resident physicians across Japan to participate in the study using the purposive and snowball sampling method. A non-anonymous web conference was held with three additional external evaluators to finalize the item list. The consensus criterion was defined as a mean response of at least 4 points on a 5-point Likert scale for each competency and EPA item and a rating of 4 or 5 points by at least 80% of the participants.

**Results:**

Consensus was achieved, with consistently high levels of agreement across panel members, on 11 competencies and 9 EPA items. Additionally, a correspondence matrix table between competencies and EPAs was developed.

**Conclusions:**

This study clarified the standardized educational outcomes as competencies in death pronouncement practice and the unit of professional practice of physicians who can perform this independently (EPAs), serving as a blueprint to aid the development of an educational model and evaluation method for clinical educational institutions and developers of medical school curriculums.

**Supplementary Information:**

The online version contains supplementary material available at 10.1186/s12909-022-03149-5.

## Background

Death pronouncement is one of the most challenging clinical practices, especially for younger physicians [1–3]. Physician-patient-family communication through the death pronouncement practice is critically important, as it potentially affects the families’ emotional and psychological wellbeing either positively or negatively (e.g., acute grief and long-term depression) [4]. This is because the death of a loved one is a critically serious life event for the family members and relatives of the deceased [4, 5]. Appropriate death pronouncement practice itself can potentially be a type of bereavement care for family members [6, 7].

Recently, better ways to deliver the death pronouncement have been actively investigated. Kusakabe et al. reported that family members responded positively toward physicians’ behaviors such as acting calmly (not rushed), having a suitable appearance for the situation, introducing themselves to the family members, and explicitly explaining the cause of death [8]. Hatano et al. revealed that bereaved caregivers did not appreciate automatic or routine pronouncement behaviors in a palliative care unit setting [9]. Mori et al., using randomized and scripted video-vignettes, found that physician behavior that was evaluated favorably by family members included five components: “waiting until the families calm themselves down, explaining that the physician has received a sign-out containing information of the patient’s condition, performing the examination respectfully, ascertaining the time of death with a wristwatch, and reassuring the families that the patient did not experience pain” [[7], p. 191–192]. Moreover, multiple educational models regarding death pronouncement practice that describe the step-by-step procedure (e.g., the GRIEV_ING model) have been proposed and investigated [10–15]. These studies have shown that compassionate and calm behavior throughout the practice is universally important regardless of the specific cultural context [7–15].

Despite the cumulative evidence of better ways to deliver the death pronouncement, a consensus has never been developed on the standardized educational outcomes as competencies in death pronouncement practice, or about the unit of professional practice who can perform this practice independently (i.e., entrustable professional activities; EPAs). Rooted in a criticism of knowledge-oriented education and a social demand to clarify standardized educational outcomes, nowadays, competency-based education that integrates knowledge, skills, and attitudes that learners need to acquire has been attracting attention [16]. Nevertheless, competencies are often theoretical and therefore difficult to teach and assess for both learners and teachers [17]. To translate competency into action, clarifying the EPAs is also widely recognized as important [17, 18]. An EPA is a unit of professional practice that can be fully entrusted to a trainee with sufficient competence to execute the activity unsupervised [18]. In daily practice, younger physicians often need to deliver the death pronouncement without mentor supervision. In Japan, it is uncommon for resident physicians to independently deliver the death pronouncement during their two-year residency period, however, senior physicians—those with over 3 years of experience—are frequently required to deliver it independently. Developing EPAs is, therefore, critical, as clarifying the kind of competencies that younger physicians need to acquire would potentially promote educational goal setting and work-based assessment, ultimately positively affecting the bereaved families’ wellbeing [19].

The aim of this study is to develop a list of the competencies and EPAs of physicians’ behaviors in death pronouncement practice, which resident physicians need to acquire by the time of completion of their residency training (postgraduate year 2).

## Methods

**Fig. 1:**
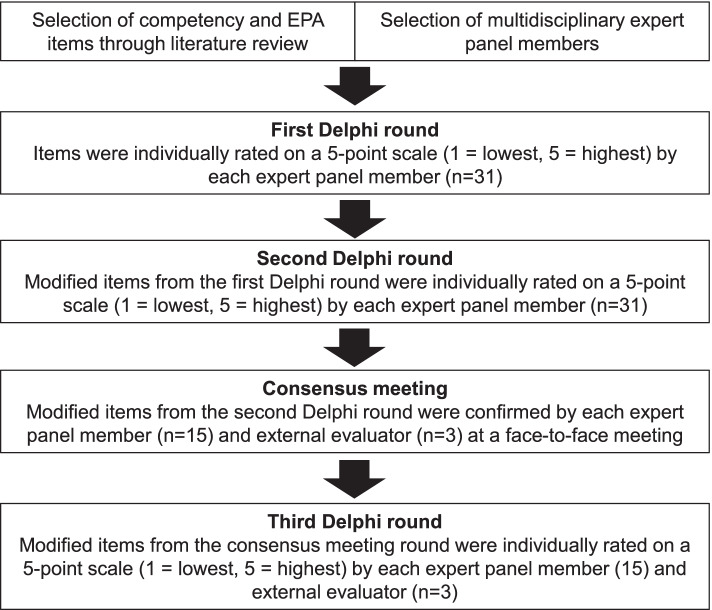
Modified Delphi technique process used to develop competencies and entrustable professional activities

Figure 1 shows the flow of this study. First, the extant literature was reviewed narratively, and an expert panel was selected using purposive and snowball sampling methods. Then, Delphi rounds were held using web technology [20–22]. Competencies and EPA items identified by the Delphi rounds were confirmed and discussed through a non-anonymous web conference between the expert panel and three external evaluators. Lastly, another Delphi round was held to finalize the competency and EPA items. The Delphi process was conducted between August 2020 and January 2021 in Japan according to the Guidance on Conducting and REporting DElphi Studies [22].

### Design

The current study was conducted using a modified Delphi method. This method is widely accepted as a scientific consensus-building method to seek experts’ opinions regarding a particular issue [20–22]. As multiple studies regarding death pronouncement have previously been reported, it was considered that interactive discussions among panel members could generate novel findings, for which the modified Delphi method was suitable compared with other techniques, including the Delphi method.

### Expert panel selection

All panel members were recruited using purposive sampling and snowball sampling [21]. The panel members were selected nationwide through consensus among the researchers to ensure heterogeneity in terms of age, organization, and years of clinical and professional experience and expertise. Since we considered that nurses play an essential role in bereavement care, before and after the delivery of the death pronouncement, and since they are considered to be essential members of multidisciplinary care teams, we included them in the Delphi survey. We recognized the importance of involving patient representatives and medical education professionals on this subject. However, as to be a specialist in death pronouncement, we thought that patient representatives and medical education professionals may not be appropriate, therefore, we decided to seek the opinions of patient representatives and medical education specialists during the consensus meeting mentioned below.

The inclusion criteria were physicians and nurses who 1) were involved in end-of-life care and death pronouncement in daily practice and 2) had experienced death pronouncement practice for 50 or more patients. The exclusion criteria were persons who were mentally unstable or had psychological conditions which made them unsuitable for participation in the research (e.g., past traumatic experiences related to bereavement).

Although panel member size for the Delphi method varies in the literature, it is generally recommended to have at least 20 members [20–22]. Considering a response rate of approximately 80% based on previous studies, the minimum recruitment number was 25 [23].

### Delphi process

First, we performed a thorough narrative literature review and developed a draft version of the competencies and EPAs item list. This was because we thought that to effectively train younger physicians, the defined competencies alone were not sufficient for on-site clinical education and that specific actions (EPAs) were also necessary [16, 24]. In other words, we thought EPA is a much easier concept for practitioners to conceptualize than invisible competencies [17]. Since this study’s focus was on physicians in a residency training program, the general ward of hospitals, where most physicians work, was the targeted setting.

Second, we constructed a web-based questionnaire using Google Forms®, posted the draft version of the item list. Prior to conducting the survey, we conducted a pilot survey with three physicians and four nurses who met the eligibility criteria, and modified the questionnaire based on its results. The panel members were asked to respond to each item using a 5-point Likert scale to provide their opinion on whether an item should be included in the list. The response options were 1 (should definitely be excluded), 2 (should be excluded), 3 (neither), 4 (should be included), and 5 (should definitely be included). In addition, there was a free text option for the panel members to make suggestions regarding any modification in wording or addition of other items. The web URL of the questionnaire was sent via e-mail to the panel members, and they were asked to answer the questionnaire independently.

Revisions to the items were made according to the panel members’ responses and discussion among researchers. Based on previous literature [20–23], and discussion within the research team, the consensus criteria to retain items from the draft list were set as 1) average of 4 points or higher on the 5-point Likert scale and 2) more than 80% of the panel members rating it as 4 or 5. When the consensus criteria had been met and no more significant comments emerged, the researchers discussed and decided more rounds were not required. To ensure anonymity and autonomous response, the list of panel members was blinded among the members and responses were assigned individual codes.

A consensus meeting was held in a non-anonymous face-to-face web conference with three additional external evaluators, including a patient representative, resident physician, and medical education professional, to assess the face validity of the list of items developed through the first and second Delphi rounds. We considered that the participation of patient representatives who underwent death pronouncement delivery, resident physicians who were the focused target of this study, and medical education professionals who would be the users of output from this study as essential for this research, and we invited them using the purposive sampling method. After the consensus meeting, the final version of the item list was developed through a third Delphi round with the attendees of the consensus meeting. Additionally, we created a draft of the matrix of competencies and EPAs, which was examined during the meeting. Then, the matrix, after minor modifications, was presented again after the third round via an e-mail to the attendees of the meeting, and was then finalized.

### Statistical analysis

Descriptive statistics were used to summarize the data using JMP version 15.0 software (SAS Institute, Cary, NC, USA).

### Ethics approval and consent to participate

The research protocol for this study was approved by University Institutional Review Board (approval number: A191100009). All panel members and external evaluators, including a patient representative, were given a sufficient explanation of the research intent in advance, and written consent to participate in the research was obtained from all participants.

## Results

### Expert panel characteristics and response rates

To ensure heterogeneity, 31 experts were recruited from across the nation (Table 1). The response rate was 31/31 (100%) in the first and second rounds among the participants in this study. Fifteen of the 31 experts were surveyed (48%), and the three external evaluating members participated in the non-anonymous web conference. The third Delphi round targeted the 18 members who attended the web consensus meeting, and 17 out of them (94%) responded.

**Table 1:** Characteristics of the Expert Panelists

	Number of expert panelists	Percentage of Total (%)	Number of consensus meeting participants	Percentage of Total (%)
Sex				
Male	15	48	8	44
Female	16	52	10	56
Age Range, y				
20–29	0	0	1	6
30–39	6	19	4	22
40–49	15	48	4	22
50–59	7	23	5	28
60–69	3	10	4	22
Occupation				
Physician	17	55	11	60
Nurse	14	45	4	22
Patient representative			1	6
Resident physician			1	6
Medical education professional			1	6
Specialty				
Physician	17	100	11	100
Internal medicine	5	29	1	9
Surgery	2	12	0	0
Palliative medicine	3	18	5	46
Oncology	1	6	1	9
Emergency medicine	2	12	1	9
Pediatrics	3	18	2	18
Obstetrics and gynecology	1	6	1	9
Nurse	14	100	4	100
Oncology	3	21	2	50
Emergency medicine	2	14	1	25
Intensive care	2	14	0	0
Geriatric	1	7	1	25
Pediatric	1	7	0	0
Palliative care	2	14	0	0
Home care	1	7	0	0
Psychiatric	2	14	0	0
Other	0	0	3	100
Clinical Experience, y				
10–19	12	39	4	22
20–29	15	48	8	44
≧30	4	13	3	17
Other	0	0	3	17
Region				
Tohoku	1	3	1	5
Kanto	16	52	10	56
Chubu	1	3	2	11
Kinki	7	23	3	17
Kyushu	5	16	2	11
Other	1	3	0	0

### First Delphi round

The results of the first Delphi round are shown in Additional file 1. Eight of the ten (80%) competency items were judged to be appropriate by consensus criteria, while two (20%) competency items led to disagreements. In the researcher meeting, minor corrections were made to the eight agreed-upon items, consistent with the panelists’ comments. Similarly, the two items for which no consensus was reached were modified according to the panelists’ comments. One of the competency items, “Recognize the history of patients’ and family members’ life and illness trajectory” was split into two items, consistent with the panel members’ comments. Seven out of the eight (87.5%) EPA items were judged to be appropriate; consensus could not be reached for one item (12.5%). For this EPA item, revisions were made as per panel members’ comments, and it was added in the second round. Additionally, as a result of free comments from the panel, one EPA item (“Explain the cause of death”) was added to the list in the second round. Thus, a total of 11 competency items and 9 EPA items were selected for the second Delphi round.

### Second Delphi round

Results of the second-round survey are shown in Additional file 2. A total of 10 of the 11 (91%) competency items were judged to be appropriate by consensus, but one (9%) competency item (competency 2; “Awareness to understand the life of patients and their families so far”) did not meet the consensus criteria. Eight of 9 (89%) EPA items were judged to be appropriate, but one (11%) EPA item (EPA 6; “Explain the cause of death”) was not. Thus, a total of 10 competency items and 9 EPA items were selected for the face-to-face web consensus meeting.

### Face-to-face web consensus meeting

As a result of discussions at the consensus meeting with four researchers, 15 panel members, and three external evaluators, competency 10, “Understand their own limitation,” and competency 12, “Reflect on the whole process of their own practice,” were added (Additional file 3).

### Third Delphi round

Results of the third-round survey are shown in Additional file 3. A total of 11 of the 12 (92%) competency items were judged to be appropriate by consensus, but 1 (8%; competency 10 “Understand their own limitation”) did not meet the consensus criteria. All 9 of the (100%) EPA items were judged to be appropriate. After the third round, the 11 competency and 9 EPA items were fixed as the final version based on the consensus (Tables 2 and 3). Two novel items that had not been referred to in previous literature, reflection and coping skills, were identified through consensus. Additionally, we created a matrix of competencies and EPAs consistent with the discussion among panel members, external evaluators, and researchers (Table 4).

**Table 2:** Competency List of Doctors’ Behaviors during Death Diagnosis

Competency Item List		Mean	Consensus Rate, n (%)
1.	Recognize patients’ illness trajectory	4.8	17 (100)
2.	Recognize the importance of a multidisciplinary approach in supporting patients and their family members	4.6	17 (100)
3.	Be aware of your own emotional wellbeing	4.5	17 (100)
4.	Cope with your own psychological distress properly	4.5	17 (100)
5.	Treat the patients and their family members with respect	4.9	17 (100)
6.	Examine patients in a correct medical manner	4.8	16 (94)
7.	Be cognizant of the distress of bereaved family members	4.5	16 (94)
8.	Communicate with compassion for family members’ emotional distress	4.7	17 (100)
9.	Be cognizant of family members’ uncertainties regarding emotion or acceptance toward the situation	4.1	16 (94)
10.	Be cognizant of the importance of behaving according to the individual	4.5	17 (100)
11.	Reflect on the entire process of your practice	4.3	16 (94)

**Table 3:** Entrustable Professional Activities in the Doctors’ Behaviors during Death Diagnosis

Entrustable Professional Activities Item List		Mean	Consensus Rate, n (%)
1.	Collect the background information of patients and their families prior to the encounter	4.5	15 (88)
2.	Share information with all clinical team members and provide bereavement care using a multidisciplinary approach	4.5	16 (94)
3.	Keep yourself neat	4.8	16 (94)
4.	Examine patients to confirm terminated vital signs	4.9	17 (100)
5.	Inform family members about bereavement in a straightforward manner	4.9	17 (100)
6.	Communicate with family members in a compassionate manner	4.7	17 (100)
7.	Discuss autopsy with attendant physician when appropriate	4.2	14 (82)
8.	Issue a death certification, sharing the contents of the document with family members	4.5	16 (94)
9.	Reflect on the entire process of your practice with mentors or colleagues when appropriate	4.4	17 (100)

**Table 4:** Mapping of Entrustable Professional Activities to a Subset of Competencies

→ Entrustable professional activities	1. Collect the background information of patients and their families prior to the encounter	2. Share information with all the members of the clinical team and provide bereavement care with a multidisciplinary	3. Keep yourself neat	4. Examine patients to confirm terminated vital signs	5. Inform the family members about the bereavement in a straightforward manner	6. Communicate with the family members in a compassionate manner	7. Discuss autopsy with the attendant physician, when appropriate	8. Issue a death certification, sharing the contents of the document with family	9. Reflect on the entire process of your practice with mentors or colleagues, when appropriate
↓ Competencies									
1. Recognize patients’ illness trajectory	〇	〇					〇	〇	
2. Recognize the importance of a multidisciplinary approach to support patients and their family members	〇	〇				〇			
3. Be aware of your emotional wellbeing		〇			〇	〇			〇
4. Cope with your psychological distress properly		〇			〇	〇			〇
5. Treat the patients and their family members with respect			〇	〇	〇	〇		〇	
6. Examine patients in a correct medical manner			〇	〇	〇		〇		
7. Be cognizant of the distress of the bereaved family members	〇	〇	〇	〇		〇		〇	
8. Communicate with compassion for family members’ emotional distress	〇		〇		〇	〇		〇	
9. Be cognizant of family members’ uncertainties regarding emotion or acceptance toward the situation	〇				〇	〇		〇	
10. Be cognitive to importance to behave according to individuality.	〇				〇	〇		〇	
11. Reflect on the entire process of your practice									〇

## Discussion

### Main findings

This study developed a list of competencies and EPAs for physicians’ behaviors in patient death pronouncement practice with a considerably high response and consensus rate. The development of the matrix of competencies and EPAs potentially helps educators to identify the competencies that learners need to acquire before performing an EPA [17, 25]. We previously published a paper regarding the practical guidelines on physicians’ behavior on the death pronouncement practice [26, 27]; the practical guidance is a subordinate concept to EPAs and provides more specific and detailed tips for clinical practice. These educational materials—competencies, EPAs, and practical guidance—would provide important insights into the development of more concrete and realistic educational models and their evaluation methods.

Eleven competencies and nine EPAs were identified, and the competencies regarding “Cognizant of the life of patients and their families” were excluded twice. The reason the competencies and EPAs converged at 11 and 9, respectively, may be that they cover a wider range of concepts and actions. For example, competencies 1 and 2 “Recognize patients’ illness trajectory” and “Recognize the importance of a multidisciplinary approach in supporting patients and their family members,” EPAs 1 and 2 “Collect the background information of patients and their families prior to the encounter” and “Share information with all clinical team members and provide bereavement care using a multidisciplinary approach” cover items from previous literature regarding “Interview the nurse: get details on the circumstances of the death, especially if the death was unexpected” and “Specific language used with families is shared (Does one say the patient has ‘died’ or ‘passed’)” [12, 28]. Moreover, competencies 5 and 6 “Treat the patients and their family members with respect” and “Be cognizant of the distress of bereaved family members” and EPA 6 “Communicate with the family members in a compassionate manner” cover items from previous literature pertaining to “Try not to say too much; this is a time to be quiet and comforting” and “Physician should not confirm death automatically or routinely” [9, 28]. The reason why the competencies regarding “Cognizant of the life of the patients and their families” were excluded twice was that to behave along with the patients’ and caregivers’ life-long context was considered to be difficult for younger physicians. Providing care during the individual’s life is believed to be important, however, it may require a considerable amount of skills and long-term clinical training [29, 30]. Therefore, for younger physicians, it would be considered of lower priority. In fact, it is pointed out that competencies and EPAs in novice learners are more limited, while those in advanced learners can be more comprehensive [31]. Therefore, EPAs for physicians at the completion of initial training, which this study focuses on, can be smaller unit of practice.

In the matrix, EPA 3 “Keep yourself neat” was classified as an expression of respect and compassion. Appropriate appearance is commonly interpreted as a type of medical professionalism [32, 33], however, in the context of death pronouncement, it may be perceived as a type of respectful and compassionate bereavement care. In fact, literature from Japan has reported on that importance of appropriate appearance and that the time of death should be confirmed using a wristwatch rather than a smartphone [7–9, 34]. Interestingly, literature from the United States also classifies “a comforting presence” as patient care [12]. Therefore, in this context, appropriate appearance may be a part of bereavement care.

### Strengths and limitations

The strength of this study lies in its method and in the expertise of the panel of experts who participated. The modified Delphi design is ideal for reaching a strong consensus, and the expertise and diversity of specialties among participants allowed us to gain a wide range of perspectives.

There are several possible limitations of this study. First, at the consensus conference and in the third Delphi round, the participation rate of the original panel members decreased. Due to the spread of COVID-19, we faced challenges in conducting on-site face-to-face meetings and adjusting the schedules of all panel members; nevertheless, we tried to get as many participants as possible. Second, the target setting of this study was general wards in hospitals and resident physicians, which can limit the external validity of the findings, such that they may not be applicable to other settings, including experienced physicians, emergency rooms, intensive care units, or home care settings. Third, this study was conducted within the cultural context of Japan. Patients, family members, and healthcare professionals in Japan tend to value relationships more than autonomy [35, 36]. Factors including religion, spirituality, or attitude toward the dead person in other cultural contexts may affect the item composition of the competencies and EPAs list. However, our list can serve as a blueprint to aid the efforts to develop an educational model and evaluation method for clinical educational institutions and developers of medical school curriculums.

### What this study adds

In our study, two novel items, reflection and coping skills, were identified through consensus. Being reflective is an important characteristic of healthcare professionals [37]. While clinical practice as a whole requires reflection, a specific clinical practice with no definitive guidelines, such as death pronouncement, may require practitioners to have a more dedicated attitude to reflect on their own practice. Adding reflection to the competencies and EPAs would emphasize this skill for younger physicians’ education and training. Moreover, since the mental health of healthcare professionals is also important, and death pronouncement can be especially burdensome for younger physicians, it is important for learners to be aware of the potential distress arising from it. This may alleviate feelings of professional loneliness and help prevent burnout.

## Conclusions

A list of competencies and EPAs for physicians’ behaviors in patients’ death pronouncement practice was developed, and two novel items, reflection and coping skills, were identified with consensus as crucial components. This list is expected to aid efforts to develop educational models based on these competencies and EPAs. Furthermore, examining the efficacy of such educational models is warranted.

## Supplementary Information

**Additional file 1.** Results of the first Delphi round in competency and entrustable professional activities items.

**Additional file 2.** Results of the second Delphi round in competency and entrustable professional activities items.

**Additional file 3.** Results of the third Delphi round in competency and entrustable professional activities items.

## Data Availability

The datasets used and/or analyzed during the current study are available from the corresponding author on reasonable request.

## Abbreviations

EPAs: Entrustable professional activities
