# Supplementary material for: Development of a list of competencies and entrustable professional activities for resident physicians during death pronouncement: a modified Delphi study
Source: BMC Med Educ. 2022 Feb 22;22:119. doi: 10.1186/s12909-022-03149-5 (PMC8861606; doi:10.1186/s12909-022-03149-5)
Supplement: Supplementary file 1 — Additional file 1. Results of the first Delphi round in competency and entrustable professional activities items. [file 12909_2022_3149_MOESM1_ESM.docx]

**Additional Table 1.** Result of 1st Delphi Round in Competency and Entrustable Professional Activities Items

| **1st Round (n=31)** | **Items** | **Mean** | **4+5, n (%)** | **Number of Comments** | **Judgement** | **Response to Comments** |
| --- | --- | --- | --- | --- | --- | --- |
| Competency 1 | Recognize the history of patients’ and family members’ life and illness trajectory | 3.8 | 19 (61) | 9 | Fail | Modify wording |
| Competency 2 | Recognize the importance of multidisciplinary collaboration | 4.5 | 28 (90) | 3 | Pass | Modify wording |
| Competency 3 | Control your emotions | 3.9 | 21 (68) | 4 | Fail | Modify wording |
| Competency 4 | Recognize the burden on yourself and take appropriate coping actions | 4.1 | 25 (81) | 5 | Pass | Modify wording |
| Competency 5 | Treat the patients and their caregivers with respect | 5 | 31 (100) | 1 | Pass | Pass |
| Competency 6 | Perform medically and legally correct medical examinations | 4.7 | 31 (100) | 3 | Pass | Modify wording |
| Competency 7 | Behave in a way that leads to care for patients and their families | 4.4 | 29 (94) | 4 | Pass | Modify wording |
| Competency 8 | Communicate while considering the family members | 4.7 | 31 (100) | 2 | Pass | Modify wording |
| Competency 9 | Be aware of family members’ uncertainties regarding emotion or acceptance toward the situation | 4.1 | 25 (81) | 7 | Pass | Modify wording |
| Competency 10 | Behave according to family members’ individuality | 4.1 | 23 (74) | 2 | Pass | Modify wording |
|  |  |  |  |  |  |  |
| EPA 1 | Collect the background information of patients and their families prior to the encounter | 4.3 | 29 (94) | 0 | Pass | Pass |
| EPA 2 | Share information with members of the clinical team and provide bereavement care using a multidisciplinary approach | 4.5 | 30 (97) | 0 | Pass | Pass |
| EPA 3 | Keep yourself neat | 4.7 | 30 (97) | 1 | Pass | Pass |
| EPA 4 | Examine patients to confirm terminated vital signs | 4.8 | 30 (97) |  | Pass | Pass |
| EPA 5 | Inform the family members about the bereavement in a straightforward manner | 4.8 | 30 (97) | 1 | Pass | Pass |
| EPA 6 | Communicate with the family members in a compassionate manner | 4.7 | 31 (100) |  | Pass | Pass |
| EPA 7 | Discuss autopsy | 3.6 | 15 (48) | 3 | Fail | Modify wording |
| EPA 8 | Issue a death certification, sharing the contents of the document with family members | 4.5 | 28 (90) | 1 | Pass | Pass |
