# Supplementary material for: Development of a list of competencies and entrustable professional activities for resident physicians during death pronouncement: a modified Delphi study
Source: BMC Med Educ. 2022 Feb 22;22:119. doi: 10.1186/s12909-022-03149-5 (PMC8861606; doi:10.1186/s12909-022-03149-5)
Supplement: Supplementary file 2 — Additional file 2. Results of the second Delphi round in competency and entrustable professional activities items. [file 12909_2022_3149_MOESM2_ESM.docx]

**Additional Table 2.** Result of 2nd Delphi Round in Competency and Entrustable Professional Activities Items

| **2nd Round (n=31)** | **Items** | **Mean** | **4+5, n (%)** | **Number of Comments** | **Judgement** | **Response to Comments** |
| --- | --- | --- | --- | --- | --- | --- |
| Competency 1 | Recognize patients’ illness trajectory | 4.4 | 31 (100) | 4 | Pass | Pass |
| Competency 2 | Be cognizant of the life of patients and their families so far | 4.0 | 23 (74) | 5 | fail | fail |
| Competency 3 | Recognize the importance of a multidisciplinary approach in supporting patients and their family members | 4.4 | 29 (94) | 2 | Pass | Pass |
| Competency 4 | Be aware of your emotional wellbeing | 4.2 | 27 (87) | 2 | Pass | Pass |
| Competency 5 | Cope with your psychological distress properly | 4.1 | 25 (81) | 3 | Pass | Pass |
| Competency 6 | Treat the patients and their family members with respect | 4.9 | 31 (100) | 1 | Pass | Pass |
| Competency 7 | Examine patients in a correct medical manner | 4.7 | 31 (100) | 2 | Pass | Pass |
| Competency 8 | Be cognizant of the distress of bereaved family members | 4.5 | 29 (94) | 4 | Pass | Pass |
| Competency 9 | Communicate with compassion for family members’ emotional distress | 4.8 | 31 (100) | 3 | Pass | Pass |
| Competency 10 | Be cognizant of family members’ uncertainties regarding emotion or acceptance toward the situation | 4.3 | 27 (87) | 4 | Pass | Pass |
| Competency 11 | Be cognizant of the importance of behaving according to the individual | 4.5 | 28 (90) | 2 | Pass | Pass |
|  |  |  |  |  |  |  |
| EPA 1 | Collect the background information of patients and their families prior to the encounter | 4.2 | 28 (90) | 1 | Pass | Pass |
| EPA 2 | Share information with all the members of the clinical team and provide bereavement care using a multidisciplinary approach | 4.5 | 30 (97) | 1 | Pass | Pass |
| EPA 3 | Keep yourself neat | 4.7 | 31 (100) | 1 | Pass | Pass |
| EPA 4 | Examine patients to confirm terminated vital signs | 4.7 | 29 (31) | 1 | Pass | Pass |
| EPA 5 | Inform the family members about the bereavement in a straightforward manner | 4.8 | 31 (100) | 1 | Pass | Pass |
| EPA 6 | Explain the cause of death | 3.8 | 21 (68) | 6 | fail | fail |
| EPA 7 | Communicate with the family members in a compassionate manner | 4.8 | 31 (100) | 3 | Pass | Pass |
| EPA 8 | Discuss autopsy with the attendant physician, when appropriate | 4.1 | 25 (82) | 3 | Pass | Pass |
| EPA 9 | Issue a death certification, sharing the contents of the document with family members | 4.5 | 27 (87) | 3 | Pass | Pass |
