# Supplementary material for: Development of a list of competencies and entrustable professional activities for resident physicians during death pronouncement: a modified Delphi study
Source: BMC Med Educ. 2022 Feb 22;22:119. doi: 10.1186/s12909-022-03149-5 (PMC8861606; doi:10.1186/s12909-022-03149-5)
Supplement: Supplementary file 3 — Additional file 3. Results of the third Delphi round in competency and entrustable professional activities items. [file 12909_2022_3149_MOESM3_ESM.docx]

**Additional Table 3.** Result of 3rd Delphi Round in Competency and Entrustable Professional Activities Items

| **3rd Round (n=17)** | **Items** | **Mean** | **4+5, n (%)** | **Judgement** |
| --- | --- | --- | --- | --- |
| Competency 1 | Recognize patients' illness trajectory | 4.8 | 17 (100) | Pass |
| Competency 2 | Recognize the importance of a multidisciplinary approach in supporting patients and their family members | 4.6 | 17 (100) | Pass |
| Competency 3 | Be aware of your emotional wellbeing | 4.5 | 17 (100) | Pass |
| Competency 4 | Cope with your psychological distress properly | 4.5 | 17 (100) | Pass |
| Competency 5 | Treat the patients and their family members with respect | 4.9 | 17 (100) | Pass |
| Competency 6 | Examine patients in correct medical manner | 4.8 | 16 (94) | Pass |
| Competency 7 | Be cognizant of the distress of bereaved family members | 4.5 | 16 (94) | Pass |
| Competency 8 | Communicate with compassion for family members’ emotional distress | 4.7 | 17 (100) | Pass |
| Competency 9 | Be cognizant of family members’ uncertainties regarding emotion or acceptance toward the situation | 4.1 | 16 (94) | Pass |
| Competency 10 | Understand your limitations | 4.1 | 13 (76) | Fail |
| Competency 11 | Be cognizant of the importance of behaving according to the individual | 4.5 | 17 (100) | Pass |
| Competency 12 | Reflect on the entire process of your practice | 4.3 | 16 (94) | Pass |
|  |  |  |  |  |
| EPA 1 | Collect the background information of patients and their families prior to the encounter | 4.5 | 15 (88) | Pass |
| EPA 2 | Share information with all the members of the clinical team and provide bereavement care using a multidisciplinary approach | 4.5 | 16 (94) | Pass |
| EPA 3 | Keep yourself neat | 4.8 | 16 (94) | Pass |
| EPA 4 | Examine patients to confirm terminated vital signs | 4.9 | 17 (100) | Pass |
| EPA 5 | Inform the family members about the bereavement in a straightforward manner | 4.9 | 17 (100) | Pass |
| EPA 6 | Communicate with the family members in a compassionate manner | 4.7 | 17 (100) | Pass |
| EPA 7 | Discuss autopsy with the attendant physician, when appropriate | 4.2 | 14 (82) | Pass |
| EPA 8 | Issue a death certification, sharing the contents of the document with family members | 4.5 | 16 (94) | Pass |
| EPA 9 | Reflect on the whole process of your own practice with mentors or colleagues, when appropriate | 4.4 | 17 (100) | Pass |
